# A Prevalent Variant in *PPP1R3A* Impairs Glycogen Synthesis and Reduces Muscle Glycogen Content in Humans and Mice

**DOI:** 10.1371/journal.pmed.0050027

**Published:** 2008-01-29

**Authors:** David B Savage, Lanmin Zhai, Balasubramanian Ravikumar, Cheol Soo Choi, Johanna E Snaar, Amanda C McGuire, Sung-Eun Wou, Gemma Medina-Gomez, Sheene Kim, Cheryl B Bock, Dyann M Segvich, Antonio Vidal-Puig, Nicholas J Wareham, Gerald I Shulman, Fredrik Karpe, Roy Taylor, Bartholomew A Pederson, Peter J Roach, Stephen O'Rahilly, Anna A DePaoli-Roach

**Affiliations:** 1 Department of Clinical Biochemistry and Medicine, University of Cambridge, Cambridge, United Kingdom; 2 Department of Biochemistry and Molecular Biology, Indiana University School of Medicine, Indianapolis, United States of America; 3 School of Clinical Medical Sciences, University of Newcastle upon Tyne, Newcastle upon Tyne, United Kingdom; 4 Department of Internal Medicine and Cellular and Molecular Physiology, Howard Hughes Medical Institute, Yale University School of Medicine, New Haven, Connecticut, United States of America; 5 Magnetic Resonance Centre, University of Nottingham, Nottingham, United Kingdom; 6 Comprehensive Cancer Centre, Duke University Medical Centre, Durham, North Carolina, United States of America; 7 Medical Research Council Epidemiology Unit, Elsie Widdowson Laboratory, Cambridge, United Kingdom; 8 Oxford Centre for Diabetes, Endocrinology and Metabolism, University of Oxford, Oxford, United Kingdom; Lund University, Sweden

## Abstract

**Background:**

Stored glycogen is an important source of energy for skeletal muscle. Human genetic disorders primarily affecting skeletal muscle glycogen turnover are well-recognised, but rare. We previously reported that a frameshift/premature stop mutation in *PPP1R3A*, the gene encoding R_GL_, a key regulator of muscle glycogen metabolism, was present in 1.36% of participants from a population of white individuals in the UK. However, the functional implications of the mutation were not known. The objective of this study was to characterise the molecular and physiological consequences of this genetic variant.

**Methods and Findings:**

In this study we found a similar prevalence of the variant in an independent UK white population of 744 participants (1.46%) and, using in vivo ^13^C magnetic resonance spectroscopy studies, demonstrate that human carriers (*n* = 6) of the variant have low basal (65% lower, *p* = 0.002) and postprandial muscle glycogen levels. Mice engineered to express the equivalent mutation had similarly decreased muscle glycogen levels (40% lower in heterozygous knock-in mice, *p* < 0.05). In muscle tissue from these mice, failure of the truncated mutant to bind glycogen and colocalize with glycogen synthase (GS) decreased GS and increased glycogen phosphorylase activity states, which account for the decreased glycogen content.

**Conclusions:**

Thus, *PPP1R3A* C1984ΔAG (stop codon 668) is, to our knowledge, the first prevalent mutation described that directly impairs glycogen synthesis and decreases glycogen levels in human skeletal muscle. The fact that it is present in ∼1 in 70 UK whites increases the potential biomedical relevance of these observations.

## Abstract 2

Stephen O'Rahilly and colleagues describe the effect of a mutation in*PPP1R3A*, present in 1.36% of participants from one UK population, that directly impairs glycogen synthesis and decreases glycogen levels in human skeletal muscle.

## Editors' Summary

**Background.:**

The human body gets the energy it needs for day-to-day living from food in a process called metabolism. However, not all the energy released by metabolism is used immediately. Some is stored in skeletal muscles as glycogen, a glucose polymer that is used during high intensity exercise. After eating, chemicals in the digestive system release glucose (a type of sugar) from food into the bloodstream where it triggers insulin release from the pancreas. Insulin instructs muscle, liver and fat cells to remove glucose from the bloodstream to keep the amount of sugar in the blood at a safe level. The cells use the glucose immediately as fuel or convert it into glycogen or fat for storage. Glycogen turnover (the depletion and replacement of glycogen stores) is tightly controlled by glycogen synthase and glycogen phosphorylase, enzymes that make and destroy glycogen, respectively. A third enzyme called protein phosphatase 1 promotes net glycogen synthesis by activating glycogen synthase and inactivating glycogen phosphorylase. The activity of protein phosphatase 1 is regulated by a family of “targeting subunits.” In muscle, one of these targeting subunits, called R_GL,_ facilitates protein phosphatase 1 action on glycogen synthase and glycogen phosphorylase.

**Why Was This Study Done?:**

Several known human genetic disorders affect the breakdown of muscle glycogen but few genetic changes (mutations) have been found that decrease the synthesis of muscle glycogen. Researchers are interested in discovering mutations that affect glycogen turnover and other aspects of metabolism because some of these may be involved in the development of diabetes, an important metabolic disorder characterized by high blood sugar levels. In this study, the researchers have investigated how a recently identified mutation in *PPP1R3A*, the gene that encodes R_GL_, affects glycogen synthesis. This mutation—*PPP1R3A FS*—was previously found in 1.36% of a UK white population. It causes the production of a short version of R_GL_ that lacks the part of the molecule that tethers R_GL_ to a cellular structure called the sarcoplasmic reticulum but leaves its glycogen binding domain intact.

**What Did the Researchers Do and Find?:**

To confirm that *PPP1R3A FS* is a common mutation in the UK white population, the researchers sequenced the gene in 744 healthy adults enrolled in the Oxford Biobank (which hopes to uncover metabolically important genetic variations by monitoring the health of a large number of 30- to 50-year-old people from whom DNA has been collected). 1.46% of these people had the *PPP1R3A FS* mutation. To examine glycogen storage in carriers of the mutation, the researchers used a technique called in vivo ^13^C magnetic resonance spectroscopy. Basal muscle glycogen levels and those reached after a meal were lower in these individuals than in people without the mutation but their blood sugar and insulin levels were normal. Finally, to examine how the mutation reduces muscle glycogen, the researchers made mice carrying the *PPP1R3A FS* mutation. Like the human carriers, these mice had less glycogen than normal in their muscles. Unexpectedly, in biochemical experiments the truncated R_GL_ protein made by the mutant mice did not bind to glycogen or co-localize with glycogen synthase. This lack of binding decreased the activity of glycogen synthase and increased the activity of glycogen phosphorylase, thus decreasing muscle glycogen.

**What Do These Findings Mean?:**

These findings identify the *PPP1R3A FS* mutation as the first prevalent mutation known to impair glycogen synthesis and to decrease glycogen levels in human skeletal muscles. They also confirm that this mutation is very common in UK whites. Although these human carriers do not report any exercise intolerance, detailed studies are needed to test whether the mutation has any effect on skeletal muscle performance. In addition, suggest the researchers, the mutation might be involved in the development of type 2 diabetes. Impaired insulin-stimulated glycogen synthesis, which is a feature of insulin-resistant muscle and liver cells, is thought to be a key event in the development of type 2 diabetes. Although some previous results indicate that the *PPP1R3A FS* mutations can sometimes predispose people to develop insulin resistance, only a large population-based study in multiple ethnic groups will reveal whether the *PPP1R3A FS* mutation has an important impact on the development of type 2 diabetes.

**Additional Information.:**

Please access these Web sites via the online version of this summary at http://dx.doi.org/10.1371/journal.pmed.0050027.

- Wikipedia has pages on metabolism and on glycogen (note that Wikipedia is a free online encyclopedia that anyone can edit; available in several languages)
- The MedlinePlus encyclopedia provides information about diabetes (in English and Spanish)
- The UK Biobank is looking for genetic variations among human populations that are associated with metabolic and other disorders
- Web sites are available with brief descriptions of the research programs of Stephen O'Rahilly and Anna DePaoli-Roach

## Introduction

The dissection of the genetic basis for interindividual variation in human metabolism is a major goal of contemporary metabolic research. Recently we identified a novel frameshift (FS) premature stop mutation in *PPP1R3A* (C1984ΔAG; stop codon 668; referred to subsequently as *PPP1R3A FS* [1]), a gene encoding the muscle-specific glycogen-targeting subunit R_GL_ (also called G_M_) of protein phosphatase 1 (PP1) [2,3]. The R_GL_ polypeptide contains an extended C-terminal tail with a short hydrophobic segment responsible for association with the sarcoplasmic reticulum [4] as well as carbohydrate- (glycogen) [5] and PP1-binding domains [6] in the N-terminal 240 residues. The latter facilitate localization of the catalytic subunit of the phosphatase (PP1c) to glycogen where it dephosphorylates glycogen synthase (GS) and glycogen phosphorylase (GP), and thereby promotes glycogen synthesis [7–9]. We have previously shown that *Ppp1r3a*-disrupted mice exhibit a 90% reduction in muscle glycogen [10], whereas R_GL_-overexpressing mice accumulate excess glycogen in muscle [11]. The *PPP1R3A FS* mutation, which was initially described in a large white kindred, results in a mutant protein lacking the long C-terminal tail including the hydrophobic segment that tethers it to the sarcoplasmic reticulum [4]. In that pedigree, severe insulin resistance was restricted to individuals who were doubly heterozygous for the *PPP1R3A FS* variant and an unlinked loss-of-function mutation in *PPARG* (AAA553T; stop codon 186), which encodes a key transcriptional regulator of adipocyte differentiation [1]. Whilst the *PPARG* variant was uniquely present in that kindred, the allelic frequency of the *PPP1R3A FS* variant was 1.36% in a population of UK whites. Although the truncated R_GL_ was shown to be mistargeted within the cell, its functional impact on glycogen synthesis was not determined. Here we sought to characterise the molecular and in vivo biological consequences of the *PPP1R3A* FS variant.

## Methods

All human studies were approved by the relevant Local Research Ethics Committees (Cambridge, Oxford, and Nottingham), and all participants provided written informed consent. The R_GL_ kin mice were generated by Cheryl Bock at the Comprehensive Cancer Center Transgenic Facility, Duke University, Durham, North Carolina, United States. All animals were maintained on a 12:12 h light–dark cycle in a temperature- and humidity-controlled facility with free access to food and water. All mouse studies were conducted in accordance with federal guidelines and were approved by the Institutional Animal Use and Care Committees of Indiana, Duke, and Yale Universities.

### Oxford Biobank and Genotyping

The Oxford Biobank consists of an age-stratified random sample of 30- to 50-year-old men and women from Oxfordshire (total population 615,200) [12]. All participants screened were classified as “white” by the researchers. Exclusion criteria included mental or physical ill health, alcohol- or drug-related problems, and significantly abnormal liver or renal function tests or anaemia. Participants attended a screening visit at the Clinical Research Unit, where blood tests were performed and basic anthropometric data recorded. DNA was stored from the visit and consent obtained to allow subsequent genotyping for genetic variants of potential metabolic importance. Primer sequences used for genotyping are available upon request.

### Determination of Muscle Glycogen Concentration in Human Participants

Six volunteers with the *PPP1R3A FS* mutation were studied (Table 1). Two of these individuals were doubly heterozygous for an additional unlinked mutation in *PPARG* and had severe insulin resistance. The results from these individuals were compared to volunteers without diabetes (*n* = 9) and participants with diet-controlled type 2 diabetes (*n* = 9) from a previous study [13]. Participants abstained from vigorous exercise and alcohol for 3 d prior to the study and each fasted for 12 h prior to the study. Baseline ^13^C measurements for muscle glycogen were taken prior to the first standard meal (*t* = 0), which consisted of 190.5 g carbohydrate, 41.0 g fat, and 28.8 g protein, totalling 1,253 kcal. Further measurements were taken at 60, 120, and 240 min, after which another standard meal of similar composition was given, followed by measurements at 300, 360, and 480 min. All glycogen measurements were performed at 3.0T, using a circular ^13^C surface coil and quadrature ^1^H coils as described previously [13]. Blood samples were taken for glucose and insulin measurements at the same times as the spectra.

**Table 1:**
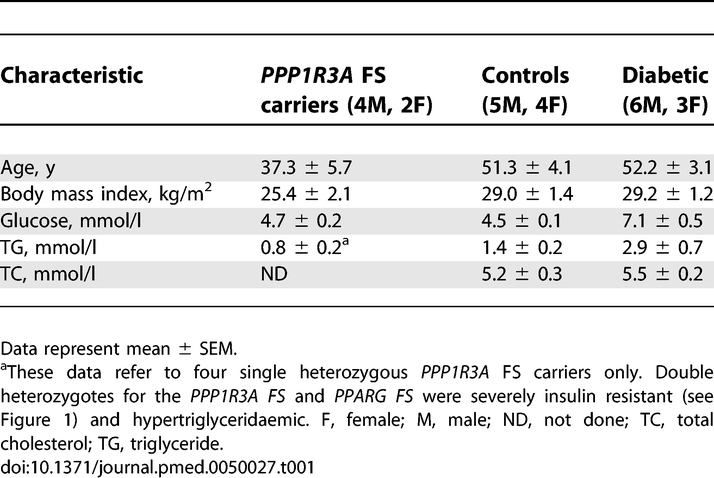
Demographic Characteristics of the Study Participants

### Generation of Mutant, R_GL_ Knock-in Mice

The *Ppp1r3a* targeting vector was assembled by PCR-amplified and restriction enzyme–digested fragments from the λ Fix 14-1-1 ES cell genomic DNA clone previously reported [2]. This clone is 13 kb and extends from 2 kb upstream of exon 2 to 7 kb downstream of the R_GL_ stop codon. The short recombination arm fragment consists of 1.2 kb of intron 3 and was inserted upstream of the 5′ LoxP site flanking the Neo cassette at the XhoI and NotI sites in the pPGKNeobpAlox2PGKDTA (Figure S1). The long recombination arm containing the FS mutation was constructed with three fragments. Part 1 was engineered to contain the FS premature stop codon found in the human participants at the corresponding position in the mouse *Ppp1r3a* by amplifying a region from 500 bp upstream of exon 4 to ∼ 1,000 bp of exon 4. The following oligonucleotide primers were used: forward 5′-TTTGCTAGCGCCGTTGACAAGTAACATGAGCCTTAT-3′ and reverse 5′-AAACCATGGTTATTCTCTCTTGATTTTCCGGGTTTCCAGAACGTTCCATTT-3′. The reverse primer encodes the altered six amino acids found in the human mutation followed by a stop codon and an NcoI site. Part 2 was a 2.2 kb NcoI-BstEII amplified fragment. Part 3 was the 4.6 kb restriction enzyme–digested BstEII-BStz171. The three fragments, totalling 8.3 kb, were inserted downstream of the 3′ LoxP site flanking the PGK-Neo gene at the NheI and EcoRV sites. Part 1 was completely sequenced to ensure that the correct mutation was introduced and that no unintended mutations were present. All junctions in the final construct were confirmed by sequencing and restriction enzyme digestion. The R_GL_ kin targeting vector also contains the diphtheria toxin A gene (PGK-DTA) for selection against random integration. The vector was linearized at the unique AhdI site and electroporated into W4 ES cells. G418-resistant cell clones were initially screened for targeting by PCR with primers ADPR 424, 5′-GCTAGACTTGATGGTAAGTGTTCTGGTTGCACAG-3′ outside of the short recombination arm and ADPR 422, 5′-CGCGAAGGGGCCACCAAAGAAGGGAGCCGGTTG-3′, in the PGK-Neo region. Positive clones were further analyzed for the presence of the mutation by utilizing ADPR 425, 5′-CCCCGGAAATCAAGAGAGAATAACCATGG-3′, which specifically recognizes the mutated sequence and not the corresponding wild type (WT), and ADPR 406, 5′-TGCGAATTCTCACCTGCCTTGAGCTTCGAGTTC-3′ located downstream. From this double screen, two positive clones were identified and correct targeting was confirmed by Southern blots of ES cell genomic DNA digested with NheI. Two fragments of 10.3 and 3.4 kb, corresponding to the WT and to the targeted allele, respectively, were detected with the 5′ probe, and 10.3 kb and 8.8 kb fragments with the 3′ probe (Figure S2A). Both clones, confirmed to be correctly targeted, were expanded and electroporated with a PGK-NLS/Cre plasmid. Resulting clones were screened by PCR for excision of the Neo cassette (efficiency 40%, 73/190) and 12 were analyzed and confirmed by Southern blot (Figure S2B). Two clones, after karyotyping, were injected into C57Bl/6J blastocysts, which were then implanted in the uterus of pseudopregnant female mice. High-percentage male chimeric mice, as judged by the agouti colour coat, were mated with C57Bl/6J mice to determine germline transmission. F1 mice were backcrossed two more times into the C57Bl/6J background, before breeding the R_GL_ kin heterozygotes to generate animals used in the study. Mice were genotyped by PCR with a pair of primers that straddle the residual loxP site as well as a pair of primers, one of which specifically hybridizes with the mutated nucleotides (Figure S2C).

### Enzyme Activity Assays

The GS and GP activities were measured as previously described [10]. Basically, powdered frozen tissue samples were homogenized in 30 volumes of buffer (50 mM Tris-HCl [pH 7.8], 10 mM EDTA, 2 mM EGTA, 100 mM NaF, 2 mM benzamidine, 0.1 mM N^α^-p-tosyl-L-lysine chloromethyl ketone, 50 mM β-mercaptoethanol, 0.5 mM PMSF, and 10 μg/ml leupeptin) using a Tissue Tearer Model 285–370 (Biospec Products) at maximal speed for 20 s. After centrifugation at 3,600*g* for 5 min, 30 μl of the supernatant was used for GS and GP assays. GS activity was determined by measuring incorporation of [^14^C]glucose from UDP-[^14^C]glucose into glycogen as described by Thomas et al. [14] in the absence or presence of 7.2 mM glucose-6-phosphate (G6P). GP activity was assayed by measuring incorporation of [^14^C]glucose from [^14^C]glucose-1-phosphate into glycogen in the absence or presence of 2 mM AMP [15]. One unit of GS is the amount of enzyme that incorporates 1 μmol/min of ^14^C-glucose from UDP-[U-^14^C]glucose into glycogen and 1 unit of GP, the amount of enzyme that incorporates 1 μmol/min of [^14^C]glucose from [U-^14^C]glucose-1-phosphate. Activity ratios represent the activity measured in the absence divided by that in the presence of the allosteric effectors G6P for GS or AMP for GP and provide an index of the phosphorylation state and hence, activity of the enzymes.

#### Other procedures.

Information about Western immunoblotting, immunoprecipitations, GST-GN pull-downs, glycogen pellets, glucose and insulin tolerance tests, and hyperinsulinemic-euglycemic clamps is included in Text S1.

### Statistical Analysis

All data are expressed as mean ± standard error of the mean (SEM). Two-tailed Student *t*-tests or one-way ANOVA plus Tukey HSD multiple comparisons were performed on data at a minimum *p* < 0.05 threshold.

## Results

### The *PPP1R3A FS* Mutation Is Prevalent in UK Whites and Impairs In Vivo Glycogen Synthesis

**Figure 1:**
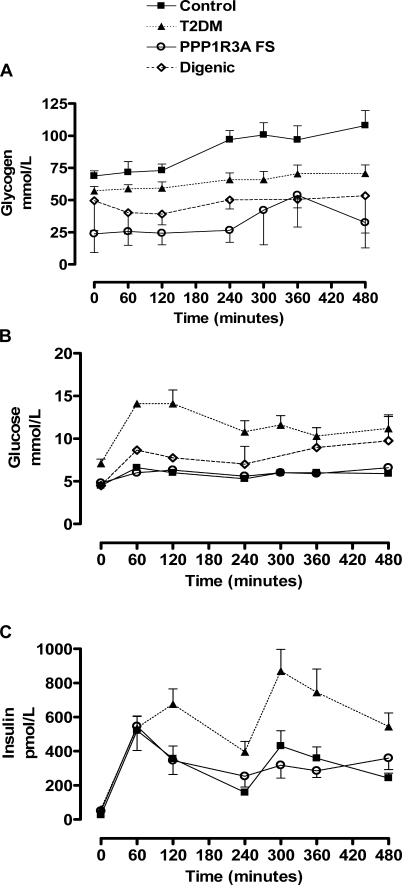
Human *PPP1R3A FS* Carrier Phenotype (A) Muscle glycogen, (B) blood glucose, and (C) insulin levels in humans with the *PPP1R3A FS* mutation before and after two standardised meals (given at 60 and 240 min). Data from *PPP1R3A FS* carriers (*n* = 4; open circles) are compared with those from volunteers without diabetes (*n* = 9; solid squares) and from participants with type 2 diabetes (T2DM; *n* = 9; filled triangles). Mean data from two digenic participants (double heterozygotes for the *PPP1R3A FS* and *PPARG FS*) are included in graphs (A) and (B). Mean fasting insulin levels from the two digenic individuals were 199, 5,744, 9,534, 1,303, 13,689, and 10,291 pmol/l, too high to include in (C), at 0, 60, 120, 240, 360, and 480 minutes, respectively.

We genotyped 744 adults without diabetes from an Oxfordshire Biobank and found that the *PPP1R3A FS* allelic frequency was 1.46%. To determine the in vivo effects of this truncated variant on skeletal muscle glycogen synthesis, ^13^C magnetic resonance spectroscopy studies were undertaken in nondiabetic volunteers of known genotype from the Oxfordshire study. Baseline muscle glycogen concentration (23.9 ± 14.7 mmol/l) was significantly lower (65%, *p* = 0.002) in *PPP1R3A FS* heterozygotes than in nondiabetic volunteers (68.9 ± 4.1 mmol/l) and even volunteers with type 2 diabetes (57.1 ± 3.6 mmol/l, *p* = 0.01) (Figure 1A) [13]. After a meal, mean glycogen concentrations increased in nondiabetic volunteers (97.1 ± 7.0 mmol/l at 240 min; *p* = 0.005). Glycogen levels peaked at 108.0 ± 11.6 mmol/l after a second meal (Figure 1A). This response was blunted in *PPP1R3A FS* carriers. Despite the significant differences in muscle glycogen levels, plasma glucose and insulin measurements were similar in nondiabetic volunteers and *PPP1R3A FS* carriers (Figure 1B and 1C). Muscle glycogen concentrations were decreased to a similar extent in two severely insulin-resistant individuals who were doubly heterozygous for the *PPP1R3A FS* variant and a *PPARG FS* loss-of-function mutation (Figure 1A) [1]. Both digenic participants were glucose intolerant and markedly hyperinsulinemic (Figure 1B and 1C).

### Mice Carrying the *PPP1R3A FS* Mutation Have Abnormal Glycogen Metabolism

**Figure 2:**
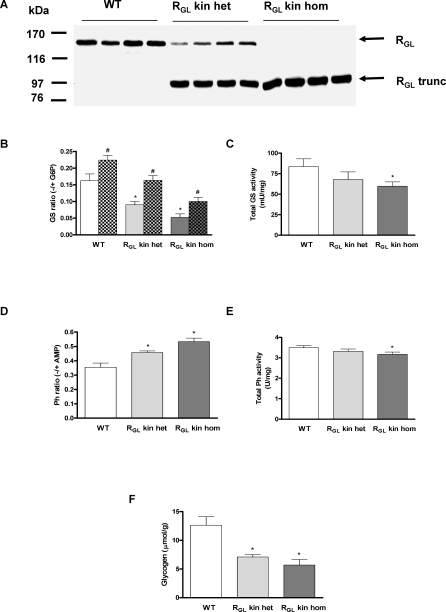
Muscle Glycogen Metabolism in R_GL_ Knock-in and Wild-Type Mice (A) R_GL_ Western blots of skeletal muscle extracts from WT, R_GL_ knock-in heterozygous (R_GL_ kin het), and R_GL_ knock-in homozygous (R_GL_ kin hom) mice. The predicted molecular weight (MW) of the truncated R_GL_ (R_GL_ trunc, 643 amino acids) is 72,000 but it migrates on SDS polyacrylamide gel electrophoresis with an apparent MW of 83,000. (B) GS activity was assayed in the absence (−) or presence (+) of G6P in extracts of skeletal muscle from WT, R_GL_ knock-in heterozygous (R_GL_ kin het), and R_GL_ knock-in homozygous (R_GL_ kin hom) mice not injected or injected intraperitoneally with 5 mU/g insulin for 10 min (plain and chequered bars, respectively). (C) Total GS activity (mU/mg) measured in the presence of 7.2 mM G6P. (D) Glycogen phosphorylase (Ph) activity was assayed in the absence (−) or presence (+) of 2 mM AMP. (E) Total glycogen phosphorylase (Ph) activity (U/mg) measured in the presence of 2 mM AMP. (F) Glycogen content in muscle. *n* = 4–9 per group; * *p* < 0.05 versus WT basal; # *p* < 0.05 insulin versus basal.

*Ppp1r3a FS* mutant mice (R_GL_ kin) were generated by introducing the human mutation, in which deletion of two base pairs results in six altered amino acids before encountering a stop codon [1], into the mouse *Ppp1r3a* locus. Therefore, a nucleotide sequence encoding the six altered amino acids and a stop codon were introduced at the corresponding position in the mouse gene by homologous recombination (Figure S1). All mice used in the studies were backcrossed three or four generations into the C57BL6/6J background. The resulting *Ppp1r3a* locus contains one *LoxP* site in intron 3 and the frameshift mutation in exon 4. The predicted truncated R_GL_ polypeptide consists of 634 residues with a Mr of 72,000. Western blotting of skeletal muscle extracts confirmed expression of the truncated R_GL_ (R_GL_ trunc) (Figure 2A), which on SDS-PAGE migrates with an apparent Mr of 83,000, higher than predicted. This altered gel migration is consistent with the properties of R_GL_, which as the full-length form also has slower electrophoretic mobility (160 kDa) than predicted (123 kDa). WT R_GL_ was decreased by ∼50% in knock-in heterozygotes and was absent in the homozygotes (Figure 2A). The seemingly stronger signal of the truncated R_GL_ protein is a reflection of the lower efficiency of electrophoretic transfer of the full-length protein. From heterozygous mouse intercrosses, WT, R_GL_ kin heterozygotes and homozygotes were born at the expected mendelian ratio (1:2:1; WT 35, heterozygotes 81, and homozygotes 39). Growth, weight, and lean and fat mass were similar in all groups of animals up to the age of 9 mo (unpublished data).

The GS −/+ G6P activity ratio, which reflects the phosphorylation and activity state of the enzyme, was significantly decreased in muscle of both heterozygous (45%) and homozygous (68%) R_GL_ kin mice (Figure 2B), whereas the GP −/+ AMP activity ratio was increased (30% and 50% respectively) (Figure 2D). Total GS and GP activities were also altered in the homozygous R_GL_ kin mice (Figure 2C and 2E). As a consequence, muscle glycogen content was significantly decreased in R_GL_ kin mice (40% and 55% reduction in heterozygotes and homozygotes, respectively) (Figure 2F). Notably, treatment with 5 mU/g insulin resulted in a similar extent of GS activation in WT, heterozygous, and homozygous knock-in mice (Δ increase 0.05–0.07; Figure 2B) indicating that neither the decreased glycogen level nor the truncation of R_GL_ have a significant effect on GS activation by insulin. A similar response was previously observed in the R_GL_ knockout mice [10]. Very low or absent muscle glycogen in R_GL_ [10] and GS knockout mice [16], respectively, results in activation of AMP kinase, increased acetyl-CoA carboxylase phosphorylation, and a metabolic switch to increase muscle fatty acid oxidation [16,17]. AMP kinase phosphorylation in the R_GL_ kin skeletal muscle revealed no detectable alterations (unpublished data), most likely because the glycogen content in these mice is significantly higher than in R_GL_ knockouts. Liver glycogen content was similar in WT and R_GL_ kin mice (unpublished data).

### The Mutant Protein Is Mistargeted in Mouse Skeletal Muscle

**Figure 3:**
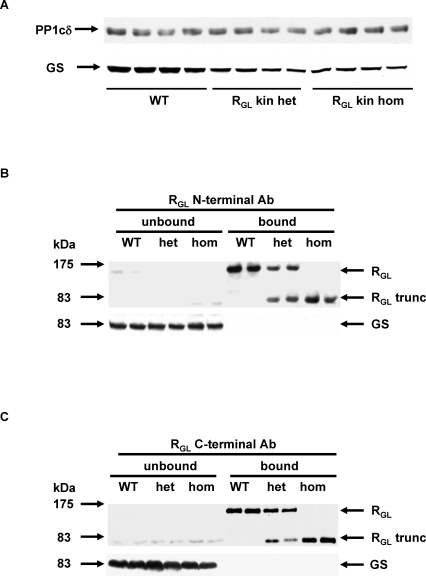
Interactions between WT R_GL_, R_GL_ Knock-In Truncated Mutant (R_GL_ trunc), and GS in Skeletal Muscle Extracts from WT, R_GL_ Knock-In Heterozygous (R_GL_ kin het) and R_GL_ Knock-In Homozygous (R_GL_ kin hom) Mice (A) Western blots of protein phosphatase-1 catalytic subunit delta (PP1cδ) and GS in muscle extracts. (B) Western blots for R_GL_ and GS following R_GL_ immunoprecipitation with a R_GL_ N-terminal antibody (Ab). (C) Western blots for R_GL_ and GS following R_GL_ immunoprecipitation with a R_GL_ C-terminal antibody (Ab).

**Figure 4:**
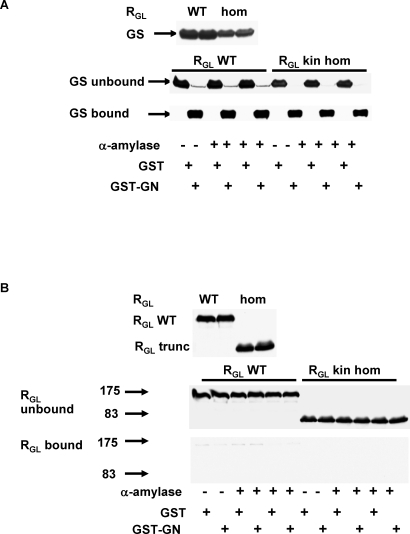
Glycogen Synthase Pull Down GS was pulled down with GST-glycogenin—GST-GN(297–333)—fusion protein before and after α-amylase digestion of glycogen in skeletal muscle extracts from wild type (R_GL_ WT) and R_GL_ knock-in homozygous (R_GL_ kin hom) mice. (A) GST-GN(297–333) pulls down almost all GS, but (B) neither the WT nor the truncated R_GL_ (R_GL_ trunc) was pulled down by GST-GN(297–333).

**Figure 5:**
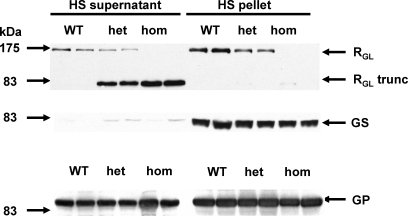
R_GL_ Western Blot Analysis of Skeletal Muscle Extracts Western blotting of high-speed supernatants and pellets of skeletal muscle extracts from WT, R_GL_ knock-in heterozygous (het), and R_GL_ knock-in homozygous (hom) mice. High speed (HS) ultracentrifugation at 100,0000*g* for 90 min was used to pellet glycogen. Western blots for R_GL_, GS, and GP were then performed on the supernatant and pellet fractions.

The truncated R_GL_ retains PP1c-, glycogen-, and putative substrate-binding motifs, all of which are located in the first 240 amino acids encoded by exon 1 [5,6,18]. Western blot analyses showed that expression of PP1cδ, the predominant isoform associated with R_GL_, was similar in all three mouse genotypes (Figure 3A). The level of GS was, however, decreased (Figure 3A), in keeping with the decreased total GS activity, most likely as a consequence of decreased glycogen, which is required for GS stability [5]. In order to gain insights into the mechanisms involved in the decreased glycogen content we assessed R_GL_ binding to GS and glycogen. Antibodies directed against both the N-terminal 262 residues and C-terminal 325-1042 amino acids of R_GL_ immunoprecipitated most of the full-length and truncated R_GL_ (note that the truncation is at residue 643), but did not coimmunoprecipitate GS, indicating that neither WT nor mutant R_GL_ binds GS (Figure 3B and 3C). This surprising result was confirmed by the reciprocal experiment in which GS was pulled down. We utilized a fusion protein (GST-GN[297–333] of GST and the residues 297–333 of glycogenin (GN), the glycogen priming protein [9], which interact with GS in two-hybrid assays and in coexpression studies [19]. While GST-GN(297–333) pulled down GS almost completely (Figure 4A), neither the WT nor the truncated R_GL_ were found associated with GS (Figure 4B). Finally, high-speed ultracentrifugation was used to precipitate glycogen and sarcoplasmic reticulum from murine muscle extracts. The vast majority of WT R_GL_ and GS, and a substantial proportion of GP were recovered in the high-speed pellet, indicating that they were all bound to glycogen (R_GL_ also binds to sarcoplasmic reticulum) (Figure 5). However, truncated R_GL_ was confined to the supernatant fraction, suggesting that the region between residue 637 and the C-terminal end of R_GL_ may contribute to glycogen binding in addition to associating with sarcoplasmic reticulum. Although we cannot exclude the possibility that the inability of the truncated R_GL_ to sediment in the high-speed pellet may be due to loss of the hydrophobic, membrane-associating domain, the fact that all the GS, which is well known to bind to glycogen, is present in the pellet argues that the ability of R_GL_ trunc to bind glycogen is decreased. In addition, after solubilising membranes with Triton X-100 in the muscle extract, we still found a significant proportion of full-length R_GL_ and all of the GS in the high-speed pellet, while the truncated R_GL_ was primarily in the supernatant (Figure S3). Ultimately, the lack of colocalization of GS and truncated R_GL_ accounts for the decreased GS activity and glycogen content in the mutant mice.

### Glucose Tolerance and Insulin Sensitivity Is Normal in R_GL_ Knock-in Mice

**Figure 6:**
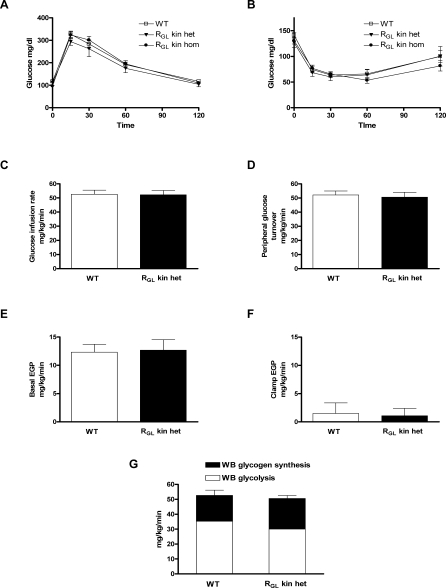
Glucose Tolerance and Insulin Sensitivity in R_GL_ Knock-in Mice (A) Glucose tolerance following intraperitoneal glucose (2 mg/g body weight) administration in WT, R_GL_ knock-in heterozygous (R_GL_ kin het), and R_GL_ kin homozygous (R_GL_ kin hom) mice. (B) Plasma glucose response to intraperitoneal insulin (0.75 mU/g body weight) in WT-, R_GL_ knock-in heterozygous (R_GL_ kin het)-, and R_GL_ knock-in homozygous (R_GL_ kin hom) mice. Peripheral and hepatic insulin sensitivity were assessed by means of hyperinsulinemic-euglycemic clamps in WT and R_GL_ kin het mice (C–G). Glucose infusion rates (C); peripheral glucose turnover (D); and basal (E) and suppressed (F) endogenous glucose production (EGP) during hyperinsulinemic-euglycemic clamps. (G) Whole body (WB) glycolysis and glycogen synthesis were measured during the clamps. Data are expressed as mean values ± SEM for 6–9 mice per treatment group.

Glucose and insulin tolerance were similar in male and female WT, R_GL_ kin heterozygotes and R_GL_ kin homozygotes (Figure 6A and 6B). As R_GL_ is only expressed in muscle, we also performed hyperinsulinemic-euglycemic clamps (with radioisotope infusions) in order to independently assess peripheral (predominantly muscle) and hepatic insulin sensitivity. Glucose infusion rates, insulin-stimulated peripheral glucose turnover, and the ability of insulin to suppress endogenous glucose production were similar in WT and R_GL_ kin heterozygous mice (Figure 6C–6F). Whole-body glycogen synthesis and glycolysis, as well as muscle insulin-stimulated glycogen synthesis, were also similar in both groups (Figure 6G).

## Discussion

Muscle glycogen is one of two major energy sources for muscle contraction, the other being fatty acids. The fuel utilized by muscle depends on factors such as the type, intensity and duration of exercise, glycogen being primarily used during short bursts of high-intensity exercise [20]. Glycogen turnover is tightly regulated by two enzymes, GS and GP. PP1 catalyzes the dephosphorylation of GS and GP, thereby activating GS, inactivating GP, and promoting net glycogen synthesis [7,8]. Its activity is regulated by a large family of targeting subunits, of which R_GL_ is the major glycogen targeting subunit in muscle [21]. Several human genetic disorders of glycogen metabolism have been described affecting muscle alone or muscle, liver, and other tissues. Until very recently, all of those affecting muscle alone impaired glycogen breakdown and caused either episodic exercise intolerance or fixed, progressive muscle weakness [20]. Kollberg et al. [22] described a consanguineous kindred in which three individuals were homozygous for premature stop mutations in *GYS1*. Affected family members presented in childhood with hypertrophic cardiomyopathy (which appeared to cause sudden death in one case) and exercise intolerance. Muscle histology revealed severe glycogen depletion and a marked increase in mitochondria-rich type 1 fibres. Glucose tolerance appeared to be normal in the single individual in whom it was assessed. This human phenotype is similar to that of the *GYS1* knockout mice [16,23]. A number of mutations in *PPP1R3A* have been identified in humans but, to date, none have been convincingly linked to in vivo alterations in glycogen metabolism [9,24–27]. We genotyped 744 nondiabetic adults from the Oxford Biobank in order to (1) assess prevalence rates of the *PPP1R3A FS* variant in an unselected population and (2) identify carriers of the variant whom we might approach for phenotyping. Prevalence figures of 1.46% are consistent with our original observation of 1.36% prevalence in a Cambridgeshire-based study [1]. Fasting and postprandial muscle glycogen levels were significantly decreased in nondiabetic *PPP1R3A FS* carriers, making this the second genetic condition known to specifically reduce muscle glycogen accumulation.

In order to precisely delineate the molecular consequences of the *PPP1R3A FS* variant we generated a knock-in mouse model, carrying the human mutation. R_GL_ kin mice were healthy, reproductively viable, and of normal body weight and fat mass, but like human carriers, had significantly decreased skeletal muscle glycogen levels. Although the truncated R_GL_ is expressed at levels similar to the WT allele and retains its capacity to bind PP1c, muscle GS activity was significantly decreased in R_GL_ kin mice and GP activity was increased. In contrast to the apparent susceptibility to degradation of an adenovirally expressed 375-residue R_GL_ fragment [28], we did not detect any evidence of degradation of our 634 amino acid FS variant. It is important to note that degradation was observed by Lerin et al. [28] only when cells were grown under nonphysiological conditions of zero glucose, a state that does not occur in humans and mice. The reduction in GS activity is nicely explained by the failure of truncated R_GL_ to colocalize with GS and therefore to direct PP1c phosphatase activity to GS. As GS is largely bound to glycogen, the inability of the truncated R_GL_ to colocalize with GS, despite the fact that it retains the glycogen-binding domain, suggests that the glycogen-binding affinity of truncated R_GL_ is decreased.

This observation is somewhat surprising, but several alternative explanations can be advanced. First, although the glycogen-binding domain in R_GL_ is essential it may not be sufficient and additional sites in the C-terminal third of the protein may also be involved in glycogen binding. Second, deletion of the C-terminal third of the protein may affect the overall protein conformation, impairing binding to glycogen. Third, it is possible that association with the sarcoplasmic reticulum is critical for glycogen binding, since our truncated R_GL_ lacks the C-terminal hydrophobic segment and cannot associate with sarcoplasmic reticulum [1]. However, the sedimentation of full-length R_GL_ and GS, but not truncated R_GL_, in the high-speed pellet after Triton X-100 solubilisation (Figure S3), argues that binding of the full-length form to glycogen is independent of sarcoplasmic reticulum association. Contrary to previous proposals [18], we also provide compelling evidence that GS does not stably interact with R_GL_ in the physiological tissue. Studies implicating a direct interaction between R_GL_ and GS utilized nonphysiological and overexpressing systems [17]. We used both coimmunoprecipitation and GST pull-down assays to show that neither full length nor truncated R_GL_ binds GS. These observations support the notion that the basic function of R_GL_ is to target PP1c to glycogen, thereby promoting dephosphorylation of glycogen bound GS and GP, a catalytic process that does not require stable interaction.

Given that the *PPP1R3A FS* variant is prevalent in UK white populations and that human physiological studies strongly implicate impaired insulin-stimulated glycogen synthesis in the pathogenesis of insulin-resistant type 2 diabetes [29,30], is there any evidence that it is a significant “diabetes genetic variant”? We originally identified the *PPP1R3A FS* mutation in a large kindred with severe insulin resistance and type 2 diabetes [1]. In that kindred only those individuals harbouring a second unlinked mutation in *PPARG* were severely insulin resistant. The *PPARG* mutation is a loss-of-function mutation, but did not manifest dominant negative activity, which is a feature of the other *PPARG* variants shown to be associated with partial lipodystrophy and severe insulin resistance [31]. In a second family, weight gain appeared to induce disproportionate insulin resistance in *PPP1R3A FS* carriers [1]. Taken together, these data suggest that carriers of the *PPP1R3A FS* variant may be predisposed to develop severe insulin resistance in the setting of adipose tissue dysfunction.

In an effort to replicate this interaction in mice, we crossed R_GL_ kin mice with PPARγ heterozygous knockouts [32] but the double heterozygosity failed to alter glucose tolerance or insulin sensitivity (Figure S4). Mouse and human muscle glycogen metabolism is very different (mouse glycogen content is much lower than that of human muscle), and the phenotype of PPARγ haploinsufficient mice is very different to that of humans with the *PPARG* FS variant. PPARγ +/− mice are protected against diet- and age-induced insulin resistance [32], whereas humans with the *PPARG FS* variant, which appears to behave as a null allele [31], manifest exaggerated hypertriglyceridaemia and insulin resistance with weight gain (unpublished data). Our original studies in Cambridgeshire volunteers did involve individuals with type 2 diabetes and controls, and the variant was significantly (*p* = 0.03) enriched in the diabetic group [1]. However, given a population prevalence of ∼1.46% and an apparently subtle phenotype, it will require very large population-based studies in multiple ethnic groups to determine whether it has a significant impact on type 2 diabetes. The *PPP1R3A FS* variant is not captured by existing fixed single nucleotide polymorphism arrays, so one cannot infer anything about its impact on diabetes risk from the recently reported type 2 diabetes genomewide association studies [33]. The FS mutation could also have functional implications for skeletal muscle performance. Anecdotally, human carriers of the *PPP1R3A FS* variant do not report exercise intolerance or muscle weakness. Studies of exercise tolerance combined with stable isotope tracing of metabolic fluxes will be needed to formally address this question in humans.

In summary, we have identified a *PPP1R3A FS* variant, which encodes a truncated protein that is mistargeted within the cell, that decreases muscle GS activity, and that increases phosphorylase activity, thereby decreasing muscle glycogen content in humans and mice. This metabolic change by itself does not alter glucose tolerance or insulin sensitivity. The findings are notable because this is a prevalent genetic mutation that clearly impairs muscle glycogen synthesis in humans. These data also demonstrate that functionally important mutations occurring at appreciable population frequencies contribute to the genetic architecture of human metabolic variation.

## Supporting Information

Figure S1 Strategy for the Generation of the Frameshift Mutant Mouse *Ppp1r3a* Locus (16 KB PDF)

Figure S2 Generation of Mice with the *Ppp1r3a* (R_GL_) Frameshift Mutation (A) Southern blots of initially targeted embryonic stem (ES) cell clones and after Cre recombinase excision of the Neo cassette. Two targeted clones were originally obtained, 1 and 2, which were analyzed by Southern blotting.
(B) After excision of the Neo cassette, six subclones from each original were analyzed by Southern blotting. Two each are shown.
(C) PCR genotyping of WT, heterozygous (het), and homozygous (hom) R_GL_ knock-in (kin) mice with a pair of primers straddling the residual loxP site or with a pair of primers that specifically recognize the frameshift mutation. AhdI, Ah; BStz171, Bs; EcoRv, Ec; NcoI, Nc; NheI, Nh; NotI, No; Truncated, Tg, XhoI, Xh.
(133 KB PDF)

Figure S3 Western Blots of Muscle Extracts Muscle extracts were prepared in the presence of 0.2% Triton X-100 to solubilise membranes. High speed (HS) ultracentrifugation at 100,000*g* for 90 min was used to pellet glycogen before Western blotting of supernatant and pellet fractions for full-length and truncated mutant (trunc) R_GL_ and GS in samples from WT, heterozygous R_GL_ knock-in (het), and homozygous R_GL_ knock-in (hom) mice.
(71 KB PDF)

Figure S4 Glucose and Insulin Tolerance Tests Glucose tolerance tests (A) and insulin tolerance tests (B) in WT, R_GL_ knock-in heterozygotes (R_GL_ kin het), PPARγ heterozygous knockouts (PPARg +/−), and doubly heterozygous mice. *n* = 7–8 per group.
(16 KB PDF)

Text S1 Supplementary Experimental Procedures (48 KB DOC)

### Accession Numbers

The GenBank (http://www.ncbi.nlm.nih.gov/) accession numbers of the genes discussed in this paper are *PPP1R3* (AF024578; Homo sapiens type-1 protein phosphatase skeletal muscle glycogen) and *Ppp1r3a* (AF309628 and AF309629; Mus musculus type 1 protein phosphatase targeting subunit RGL/ GM gene).

## Abbreviations

AMP: adenosine monophosphate
FS: frameshift
G6P: glucose 6-phosphate
GN: glycogenin
GP: glycogen phosphorylase
GS: glycogen synthase
PP1c: phosphoprotein phosphatase 1 catalytic subunit
*PPP1R3A*: gene encoding the phosphoprotein phosphatase 1 muscle-specific glycogen targeting subunit R_GL_ (also called G_M_)
R_GL_ kin: R_GL_ knock-in
SEM: standard error of the mean
WT: wild type.
