# Correction: A Prevalent Variant in *PPP1R3A* Impairs Glycogen Synthesis and Reduces Muscle Glycogen Content in Humans and Mice

**DOI:** 10.1371/journal.pmed.0050246

**Published:** 2008-12-23

**Authors:** David B Savage, Lanmin Zhai, Balasubramanian Ravikumar, Cheol Soo Choi, Johanna E Snaar, Amanda C McGuire, Sung-Eun Wou, Gemma Medina-Gomez, Sheene Kim, Cheryl B Bock, Dyann M Segvich, Bhavana Solanky, Dinesh Deelchand, Antonio Vidal-Puig, Nicholas J Wareham, Gerald I Shulman, Fredrik Karpe, Roy Taylor, Bartholomew A Pederson, Peter J Roach, Stephen O'Rahilly, Anna A DePaoli-Roach

Correction for:

Savage DB, Zhai L, Ravikumar B, Choi CS, Snaar JE, et al. (2008) A prevalent variant in *PPP1R3A* impairs glycogen synthesis and reduces muscle glycogen content in humans and mice. PLoS Med 5(1): e27. doi:10.1371/journal.pmed.0050027

One of the corresponding authors, Stephen O'Rahilly, has contacted us to request that two Ph.D. students at the time of the research—Dr. Bhavana Solanky and Dr. Dinesh Deelchand—be listed as authors on this paper for their contribution on the work on the human participants undertaken at the Sir Peter Mansfield Magnetic Resonance Centre in Nottingham, United Kingdom.

All of the other authors listed on this paper agree to this change in the authorship. We have contacted both Dr. Bhavana Solanky and Dr. Dinesh Deelchand, who have confirmed that it is appropriate for them to be listed as authors on the paper and that they have no competing interests.

The author contributions section of this manuscript should be corrected as follows:

Mouse studies were designed and performed by DBS, LZ, CSC, ACG, S-EW, GM-G, SK, CBB, DMS, AV-P, GIS, BAP, PJR, and AADP-R. Human studies were designed and performed by DBS, BR, JES, BS, DD, NJW, FK, RT, and SOR. The manuscript was prepared by DBS, SOR, and AADP-R, and seen by all authors.

