# Supplementary material for: A Prevalent Variant in PPP1R3A Impairs Glycogen Synthesis and Reduces Muscle Glycogen Content in Humans and Mice
Source: PLoS Med. 2008 Jan 29;5(1):e27. doi: 10.1371/journal.pmed.0050027 (PMC2214798; doi:10.1371/journal.pmed.0050027)
Supplement: Figure S1 — (16 KB PDF) [file pmed.0050027.sg001.pdf]

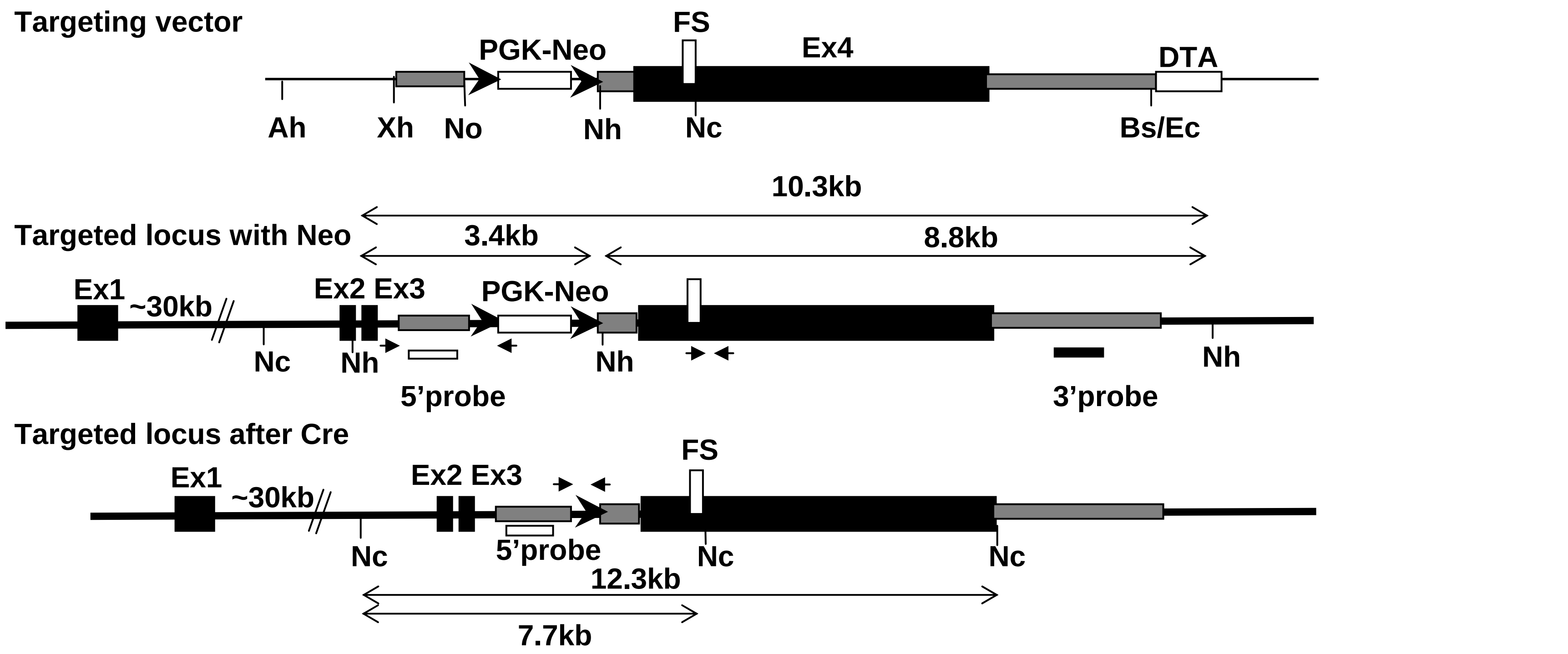

Human PPP1R3A

WT: 1980 AGTCAGGGAAAATCAAGAGAGAATAAG
WT: 660 Ser Gln Gly Lys Ser Arg Glu Asn Lys

FS: 1980 AGTCGGAAAATCAAGAGAGAATAAGAC
FS: 660 Ser Arg Lys Ile Lys Arg Glu Stop 668

Mouse PPP1R3A

WT: 1908 ACCCAGTCAGAAACAAGTGAGACTCAA
WT: 636 Thr Gln Ser Glu Thr Ser Glu Thr Glu

FS: 1908 ACCCGGAAAATCAAGAGAGAATAAGACCATGG
FS: 636 Thr Arg Lys Ile Lys Arg Glu Stop 643
