# Supplementary figures and images for: A Prevalent Variant in PPP1R3A Impairs Glycogen Synthesis and Reduces Muscle Glycogen Content in Humans and Mice
Source: PLoS Med. 2008 Jan 29;5(1):e27. doi: 10.1371/journal.pmed.0050027 (PMC2214798; doi:10.1371/journal.pmed.0050027)

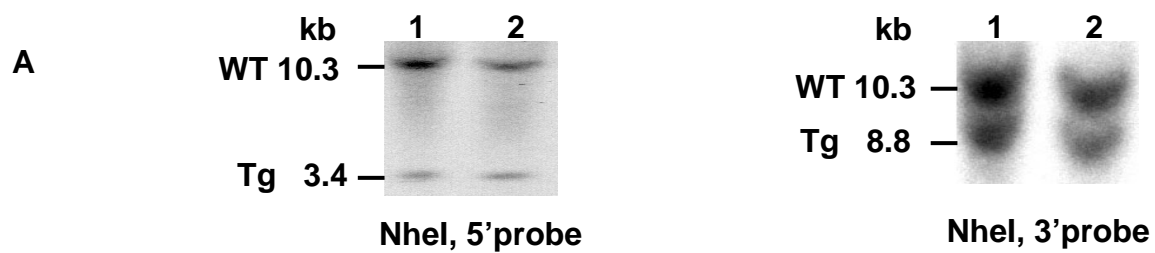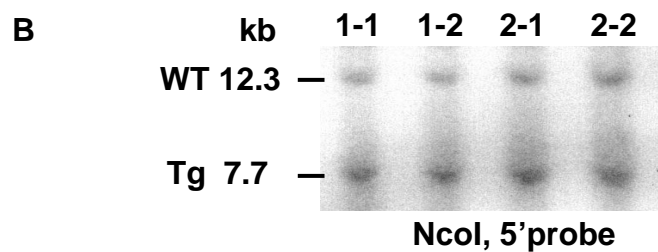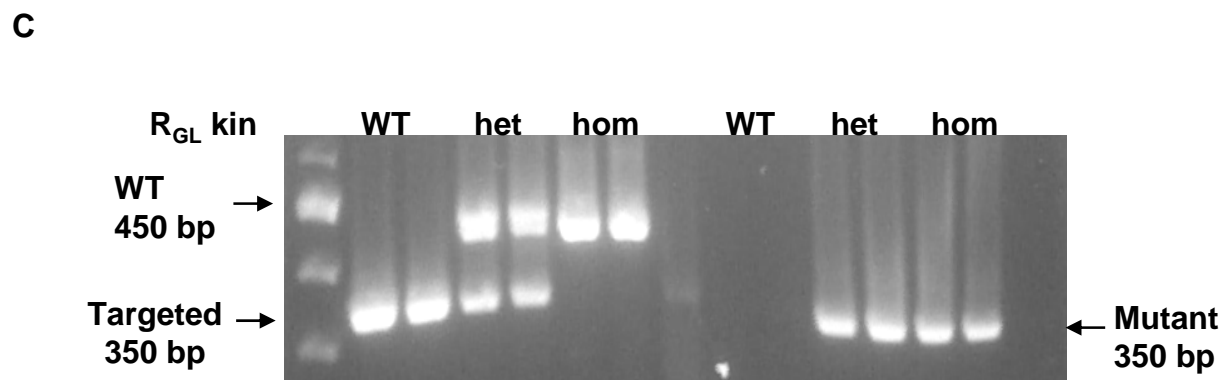

Supplement: Figure S2 — (A) Southern blots of initially targeted embryonic stem (ES) cell clones and after Cre recombinase excision of the Neo cassette. Two targeted clones were originally obtained, 1 and 2, which were analyzed by Southern blotting. (B) After excision of the Neo cassette, six subclones from each original were analyzed by Southern blotting. Two each are shown. (C) PCR genotyping of WT, heterozygous (het), and homozygous (hom) RGL knock-in (kin) mice with a pair of primers straddling the residual loxP site or with a pair of primers that specifically recognize the frameshift mutation. AhdI, Ah; BStz171, Bs; EcoRv, Ec; NcoI, Nc; NheI, Nh; NotI, No; Truncated, Tg, XhoI, Xh. (133 KB PDF) [file pmed.0050027.sg002.pdf]

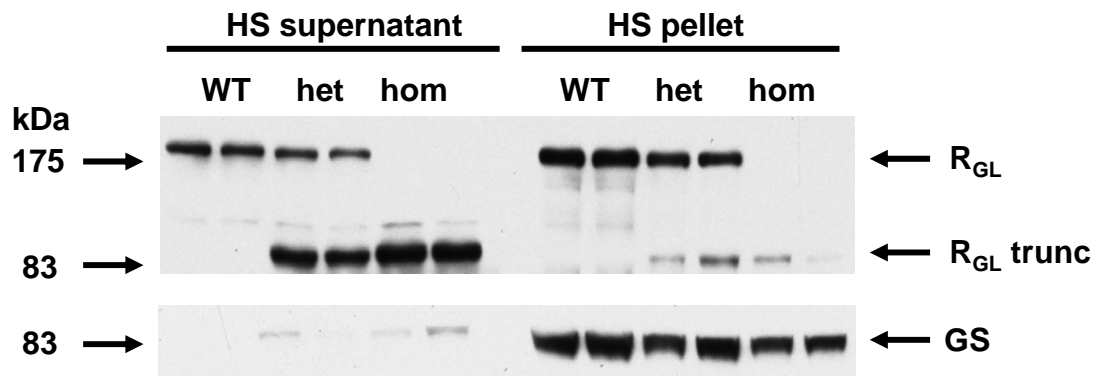

Supplement: Figure S3 — Muscle extracts were prepared in the presence of 0.2% Triton X-100 to solubilise membranes. High speed (HS) ultracentrifugation at 100,000g for 90 min was used to pellet glycogen before Western blotting of supernatant and pellet fractions for full-length and truncated mutant (trunc) RGL and GS in samples from WT, heterozygous RGL knock-in (het), and homozygous RGL knock-in (hom) mice. (71 KB PDF) [file pmed.0050027.sg003.pdf]

A

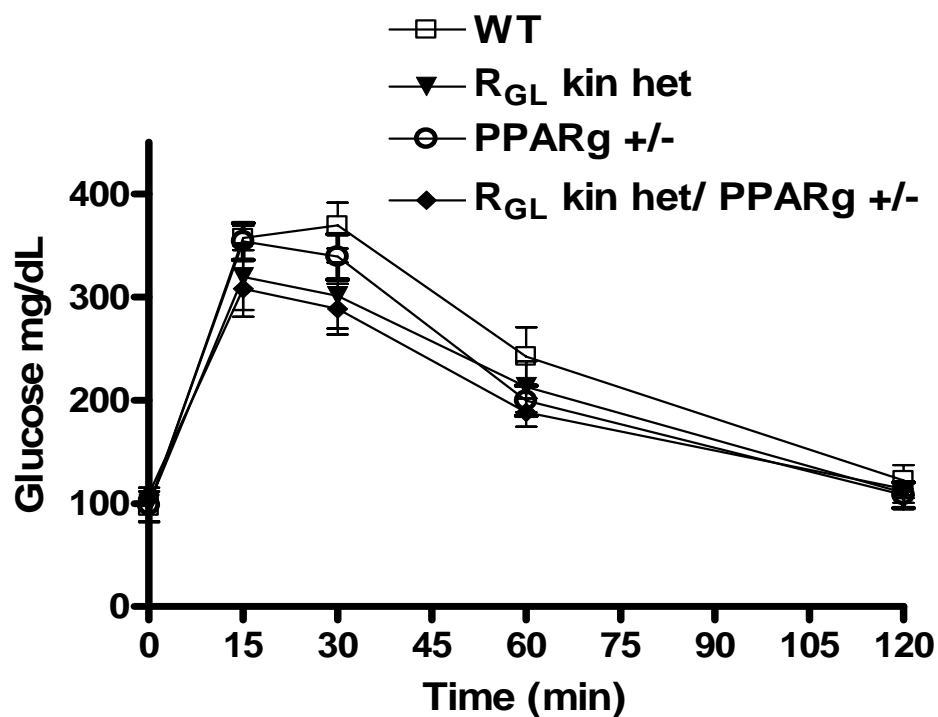

B

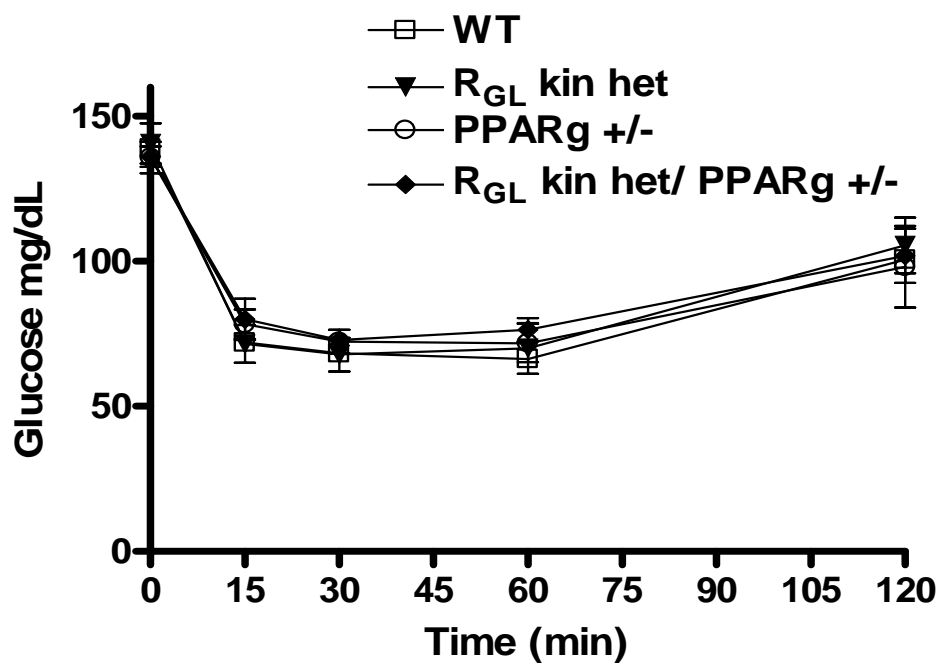

Supplement: Figure S4 — Glucose tolerance tests (A) and insulin tolerance tests (B) in WT, RGL knock-in heterozygotes (RGL kin het), PPARγ heterozygous knockouts (PPARg +/−), and doubly heterozygous mice. n = 7–8 per group. (16 KB PDF) [file pmed.0050027.sg004.pdf]
