# Supplementary material for: A Prevalent Variant in PPP1R3A Impairs Glycogen Synthesis and Reduces Muscle Glycogen Content in Humans and Mice
Source: PLoS Med. 2008 Jan 29;5(1):e27. doi: 10.1371/journal.pmed.0050027 (PMC2214798; doi:10.1371/journal.pmed.0050027)
Supplement: Text S1 — (48 KB DOC) [file pmed.0050027.sd001.doc]

# Supplementary experimental procedures

**Immunoprecipitation, pull-down, glycogen pellets and Western blot analyses**

Powdered frozen muscle samples were homogenized in 10 volumes of a buffer consisting of 50 mM Tris/HCl, pH 7.5 (25°C), 0.5 mM EDTA, 2 mM EGTA, 0.1% Triton X-100, 0.1 mM N^α^-p-tosyl-L-lysine chloromethyl ketone, 2 mM benzamidine, 0.5 mM PMSF and 10 μg/ml leupeptin with a Tissue Tearer Model 285-370 (Biospec Products Inc.) at maximal setting for 20 seconds (s). The homogenates were centrifuged at 3600 x g for 10 minutes (min). The resultant supernatants were used for various analyses. For immunoprecipitations, after pre-clearing with protein-A agarose, 100 μl of extracts were incubated for 2 hours (hrs) with either affinity purified antibodies against the mouse R_GL_ 1-262 N-terminal residues or with our recently produced anti-serum against the R_GL_ C-terminal (325-1042) residues. The immuno-complexes were harvested with protein-A agarose, washed three times and eluted by boiling in SDS. Equivalent amounts, based on the protein concentration of the extracts, were separated on SDS-PAGE. The proteins on the gels were transferred to nitrocellulose membranes, which were then incubated with antibodies against GS and the N-terminal region of R_GL_. Binding of the antibody was detected by horseradish peroxidase-conjugated secondary antibody and enhanced chemiluminescence. For the pull-down experiments, 200 μl extracts were incubated for 1hr at 30^o^C either with GST-glycogenin (GN297-333) fusion protein that has been shown to interact with GS [1] or GST alone, in the absence and presence of 0.1mg/ml α-amylase to digest the glycogen. Complexes were harvested by glutathione-agarose and after three washes were eluted by boiling in SDS. Tissue extracts, unbound and bound samples were analyzed by Western immunoblotting as described above, utilizing anti-GS and anti-R_GL_ antibodies. Preparation of glycogen pellets was performed by subjecting tissue extracts to 100,000 x g centrifugation for 90 min. Pellets were resuspended in the original volume of homogenization buffer and extracts, high-speed supernatant and pellets were analyzed by Western blotting with antibodies against GS, R_GL_, AMP-activated protein kinase (AMPK) and phospho-AMPK.

Antibodies to GS and GP were generously provided by John Lawrence, Jr. (University of Virginia, Charlottesville) and Gerald Carlson (University of Missouri-Kansas City, Kansas City, Missouri), respectively. The antibodies to the N-terminal region of R_GL_ were as previously described [2], except that they were affinity purified and anti-PP1cδ antibodies were raised in rabbit against residues 302-322 of the protein (AKYQYGGLNSGRPVTPPRTAN) and affinity purified. The R_GL_325-1042 antibodies were produced in rabbit against recombinant protein and the GST-glycogenin (297-333) fusion protein was produced in *E. coli* [1]. Antibodies to AMPK and phospho-AMPK were from Cell Signaling Technology, Inc., Beverly, MA.

**Determination of glycogen content in mouse tissues**

Glycogen content was measured as previously described [2]. Samples of frozen tissues (30-50 mg) were hydrolyzed in 0.3 ml of 30% (w/v) KOH solution in a boiling water bath for 30 min. At 10 min and 20 min of the incubation, tubes were shaken by hand to facilitate the digestion. After cooling to room temperature, 0.1 ml of 1M Na_2_SO_4_ and 0.8 ml ethanol were added, the samples were boiled again for 2 min to facilitate precipitation of glycogen and then centrifuged at 10,000 x g for 15 min. The glycogen pellet was dissolved in 0.2 ml of water and two additional ethanol precipitations were performed. The final pellet was dried and dissolved in 0.2 ml of 0.3 mg/ml amyloglucosidase in 0.2 M sodium acetate buffer pH 4.8 and incubated for 3 h at 40^o^C. The reaction mixture was then diluted 2-fold with water. To determine glucose concentration, 10 μl of the diluted sample was added to 0.3 ml of the glucose assay solution, which contains 0.3 M triethanolamine/KOH, pH 7.5, 2 mM ATP, 4 mM MgCl_2_, 0.9 mM β-NADP, and 2 μg/ml G-6P dehydrogenase. The absorbance at 340 nm was determined before and after addition of 3 U/ml hexokinase and incubation for 30 min at room tempeature. Glycogen content was expressed as μmol of glucosyl units/g wet weight.

**Glucose and insulin tolerance tests**

For glucose tolerance tests, mice were fasted for 16 hrs. Glucose (2 mg/g body weight) was administered intraperitoneally. Blood samples were collected from the tail at various times and blood glucose was measured using a Glucometer Elite (Ames). For insulin tolerance tests, 6 hr fasted animals were injected intraperitoneally with 0.75 munit/g of human insulin (Humulin, Eli Lilly and Com). Blood glucose was monitored as for the glucose tolerance test. The PPARγ+/- mice were kindly provided by Dr. Ron Evans (The Salk Institute for Biological Studies, La Jolla, California).

**Hyperinsulinemic-euglycemic clamp studies**

Male mice 4-5 month of age were used. Seven days prior to the hyperinsulinemic-euglycemic clamp studies, an indwelling catheter was placed into the right internal jugular vein extending to the right atrium. After an overnight fast, [3-^3^H]-glucose (HPLC purified; Perkin Elmer, Boston, MA USA) was infused at a rate of 0.05 μCi/min for 2 hours to assess the basal glucose turnover. Following the basal period, the hyperinsulinemic-euglycemic clamp was conducted for 120 min with a primed/continuous infusion of human insulin (3 mU/kg/min infusion) (Novo Nordisk, Princeton, NJ) and a variable infusion of 20% dextrose to maintain euglycemia (~120 mg/dl). [3-^3^H]-glucose was infused at a rate of 0.1 μCi/min throughout the clamp. A bolus of 2-deoxy-D-[1-^14^C]glucose (Perkin Elmer, Boston, MA) was injected 75 minutes into the clamp to estimate the rate of insulin-stimulated tissue glucose uptake. At the end of the clamp, mice were anaesthetized with pentobarbital sodium injection and all tissues were taken within 4 minutes, frozen immediately using liquid N_2_-cooled aluminum tongs, and stored at –80°C for subsequent analysis.

**Biochemical analysis and calculations**

Plasma glucose was analyzed during the clamps using 10 μl plasma by a glucose oxidase method on a Beckman glucose analyzer II (Beckman, Fullerton, CA). Plasma insulin was measured by radioimmunoassay using a kit from Linco Research (St. Charles, MO). Plasma fatty acid concentrations were determined using an acyl-CoA oxidase based colorimetric kit (Wako Pure Chemical Industries, Osaka, Japan). For the determination of plasma ^3^H-glucose, plasma was deproteinized with ZnSO_4_ and Ba(OH)_2_, dried to remove ^3^H_2_O, resuspended in water, and counted in scintillation fluid (Ultima Gold, Perkin Elmer, Boston MA) on a Beckman scintillation counter. Rates of basal and insulin-stimulated whole-body glucose turnover were determined as the ratio of the [3-^3^H]-glucose infusion rate (disintegrations per minute [dpm] per minute) to the specific activity of plasma glucose (dpms per mg) at the end of the basal period and during the final 30 min of the clamp experiment, respectively. Endogenous glucose production (EGP) was determined by subtracting the glucose infusion rate from the rate of total glucose appearance. The plasma concentration of ^3^H_2_O was determined by the difference between ^3^H counts without and with drying and whole body glycolysis was calculated from the rate of increase in plasma ^3^H_2_O concentration, determined by linear regression of the measurements at 80, 90, 100, 110, and 120 min [3]. Whole body glycogen synthesis was estimated by subtracting whole body glycolysis from whole body glucose uptake, assuming that glycolysis and glycogen synthesis account for the majority of insulin-stimulated glucose uptake [4]. Glucose uptake and glycogen synthesis in individual muscles were calculated from muscle ^14^C-2-DG-6-P content and ^3^H incorporation into muscle glycogen as previously described [3]. For the determination of muscle ^14^C-2-DG-6-phosphate (2-DG-6-P) content, muscle samples were homogenized, and the supernatants were subjected to an ion-exchange column to separate 2-DG-6-P from 2-DG as previously described [5]. The radioactivity of ^3^H in muscle glycogen was determined by digesting muscle samples in KOH and precipitating glycogen with EtOH as previously described [4].

**Supplementary references**

1. Skurat AV, Dietrich AD, Roach PJ (2006) Interaction between glycogenin and glycogen synthase. Arch Biochem Biophys 456: 93-97.

2. Suzuki Y, Lanner C, Kim JH, Vilardo PG, Zhang H, et al. (2001) Insulin control of glycogen metabolism in knockout mice lacking the muscle-specific protein phosphatase PP1G/RGL. Mol Cell Biol 21: 2683-2694.

3. Youn JH, Buchanan TA (1993) Fasting does not impair insulin-stimulated glucose uptake but alters intracellular glucose metabolism in conscious rats. Diabetes 42: 757-763.

4. Rossetti L, Giaccari A (1990) Relative contribution of glycogen synthesis and glycolysis to insulin-mediated glucose uptake. A dose-response euglycemic clamp study in normal and diabetic rats. J Clin Invest 85: 1785-1792.

5. Ohshima K, Shargill NS, Chan TM, Bray GA (1984) Adrenalectomy reverses insulin resistance in muscle from obese (ob/ob) mice. Am J Physiol 246: E193-197.
